# Fine-Scale Skeletal Banding Can Distinguish Symbiotic from Asymbiotic Species among Modern and Fossil Scleractinian Corals

**DOI:** 10.1371/journal.pone.0147066

**Published:** 2016-01-11

**Authors:** Katarzyna Frankowiak, Sławomir Kret, Maciej Mazur, Anders Meibom, Marcelo V. Kitahara, Jarosław Stolarski

**Affiliations:** 1 Institute of Paleobiology, Polish Academy of Sciences, Twarda 51/55, PL-00-818 Warsaw, Poland; 2 Institute of Physics, Polish Academy of Sciences, Lotników 32/46, PL-02-668 Warsaw, Poland; 3 Department of Chemistry, University of Warsaw, Pasteura 1, PL-02-093 Warsaw, Poland; 4 Laboratory for Biological Geochemistry, School of Architecture, Civil and Environmental Engineering, Ecole Polytechnique Fédérale de Lausanne (EPFL), CH-1015 Lausanne, Switzerland; 5 Center for Advanced Surface Analysis, Institute of Earth Sciences, Université de Lausanne, CH-1015 Lausanne, Switzerland; 6 Departamento de Cięncias do Mar, Universidade Federal de SăoPaulo, Campus Baixada Santista, 11030–400 Santos, Brasil; University of Bologna, ITALY

## Abstract

Understanding the evolution of scleractinian corals on geological timescales is key to predict how modern reef ecosystems will react to changing environmental conditions in the future. Important to such efforts has been the development of several skeleton-based criteria to distinguish between the two major ecological groups of scleractinians: zooxanthellates, which live in symbiosis with dinoflagellate algae, and azooxanthellates, which lack endosymbiotic dinoflagellates. Existing criteria are based on overall skeletal morphology and bio/geo-chemical indicators—none of them being particularly robust. Here we explore another skeletal feature, namely fine-scale growth banding, which differs between these two groups of corals. Using various ultra-structural imaging techniques (e.g., TEM, SEM, and NanoSIMS) we have characterized skeletal growth increments, composed of doublets of optically light and dark bands, in a broad selection of extant symbiotic and asymbiotic corals. Skeletons of zooxanthellate corals are characterized by regular growth banding, whereas in skeletons of azooxanthellate corals the growth banding is irregular. Importantly, the regularity of growth bands can be easily quantified with a coefficient of variation obtained by measuring bandwidths on SEM images of polished and etched skeletal surfaces of septa and/or walls. We find that this coefficient of variation (lower values indicate higher regularity) ranges from ~40 to ~90% in azooxanthellate corals and from ~5 to ~15% in symbiotic species. With more than 90% (28 out of 31) of the studied corals conforming to this microstructural criterion, it represents an easy and robust method to discriminate between zooxanthellate and azooxanthellate corals. This microstructural criterion has been applied to the exceptionally preserved skeleton of the Triassic (Norian, ca. 215 Ma) scleractinian *Volzeia* sp., which contains the first example of regular, fine-scale banding of thickening deposits in a fossil coral of this age. The regularity of its growth banding strongly suggests that the coral was symbiotic with zooxanthellates.

## Introduction

The ecological success of modern, shallow water reef-building corals is, for a large part, attributed to their symbiosis with *Symbiodinium* spp. dinoflagellates (zooxanthellae). On one hand, endosymbiotic zooxanthellae support the metabolism of their coral hosts by delivery of photosynthesis-derived nutrients through rapid translocation processes [1,2]. This allows corals to thrive in nutrient-poor tropical waters [3,4]. Moreover, the dinoflagellates have been shown to indirectly support coral biomineralization [4], and thus contribute to the phenomenon of light enhanced skeletal formation (light-enhanced calcification [5–9]). On the other hand, symbiont dinoflagellates find protection and moderated light conditions in the coral tissue and receive metabolically recycled products of coral metabolism [10,11].

Symbiosis between scleractinians and zooxanthellae has played an important role in evolution of reef ecosystems since Late Triassic [12,13]. Reef ecosystem collapse during mass extinctions have been linked with loss of dinoflagellate algae (bleaching), while subsequent recoveries have been linked to the renewal, or recovery of the symbiotic relationship [14–16]. Understanding how this symbiotic relationship has evolved in the past is crucial for paleoenvironmental reconstructions, for interpreting fluctuations in reef formation rates, and form part of the basis for long-term predictions of the fate of present day reefs under changing environmental conditions.

The main challenge in identification of coral symbiosis in the fossil record is that direct evidence for the presence or absence of endosymbionts is not preserved. Therefore indirect evidence (proxies) of their presence (or absence) must be developed. For example, it is commonly assumed that due to light requirements for photosynthesis, the presence of zooxanthellae is restricted to the shallow photic zone, whereas azooxanthellate corals that do not have the same light constraints can live in deep-water environments. However, due to special adaptations, e.g., fluorescent pigments in *Leptoseris fragilis*, some symbiotic corals can live nearly at the edge of the photic zone, below 200 meters [17–19]. On the other hand, a number of azooxanthellate species like *Tubastraea micranthus* or *Astroides calycularis* inhabit exclusively shallow-waters [20].

In order to differentiate symbiotic and asymbiotic corals, several skeleton-based criteria have been proposed, all of which are based on comparisons with skeletal features of their modern representatives:

(1) Overall skeletal morphology. Coates & Jackson [21] suggested that the presence of zooxanthellae is reflected in the morphology of coral skeleton. Symbiotic corals often have corallites of small size and form highly integrated colonies (e.g., meandroid or thamnasterioid colonies) with large parts of the surface covered by coenosarc (interpolypoidal tissue) [14]. On the other hand, species lacking zooxanthellae preferentially form solitary coralla or poorly integrated colonies (i.e. with little or no connecting tissue) and have larger corallites; e.g., phaceloid growth forms [22]. However, there are several exceptions to this criterion. For example, the symbiotic corals *Fungia* and *Cynarina* form large, solitary coralla, and some asymbiotic-corals form well-integrated colonies composed of relatively small corallites: e.g. *Astrangia poculata* (cerioid) or *Astroides calycularis* (plocoid) [23–26]. This morphological criterion alone is therefore not enough to robustly distinguish fossil symbiotic vs. non-symbiotic corals and must be complemented by additional indicators.
(2) Geochemical criteria. Skeletons of symbiotic and asymbiotic corals exhibit differences in their isotopic signatures [27]. Quite strong and positive correlations between skeletal carbon (δ^13^C) and oxygen (δ^18^O) isotopic compositions are observed for asymbiotic corals, whereas such correlations are much weaker or absent in symbiotic coral skeletons [28,29]. In addition, intraskeletal organic matrix (OM) extracted from skeletons of symbiotic corals exhibits different nitrogen isotopic composition (δ^15^N) compared with OM from asymbiotic coral species [30].
(3) Biochemical criteria. Zooxanthellate and azooxanthellate corals differ in composition of intra-skeletal organic matrix [31,32,33]. Relative concentration of some amino acids and sugars differs depending on presence or absence of photosymbionts [31].

Although, both bio- and geochemical features hold great potential to identify symbiosis, their application to fossil skeletons is reasonable only with exceptionally well preserved samples. This is extremely rare because both the aragonite skeleton and its organic matrix are highly vulnerable to secondary alteration [34,35]. Even skeletons considered ‘exceptionally well preserved’ might in fact contain nanometer scale diagenetic features that alter the original chemical and isotopic compositions [36].

(4) Microstructural criteria. In contrast, microstructural features of coral skeletons can be observed in fossil corals that are not entirely recrystallized, which includes many species recovered from sediments as old as Triassic [37,38]. It has been suggested that symbiotic and asymbiotic corals can be distinguished on the basis of microstructure of thickening deposits (traditionally referred to as fibers) [39]. Because skeletal growth of symbiotic corals is affected by the diurnal, cyclical activity of photosymbionts, one could expect that their skeletal growth increments will be characterized by a high degree of regularity. On the other hand, growth increments formed by asymbiotic corals, which are not (or less directly) affected by the diurnal cycle, might be less regular.

To date, a microstructural criterion based on skeletal growth increments has not been systematically developed or tested. Moreover, basic aspects of fine-scale growth banding, such as correlation with geochemical observations, have not been satisfactorily documented and explained. Here we present systematic observations of structural characteristics of micro-scale banding of a large suite of extant symbiotic and asymbiotic corals. Examination of samples from both shallow- and deep-water settings, and from geographically diverse localities lends credibility to the use of a microstructural criterion for distinguishing between zooxanthellate vs. azooxanthellate corals and application of this criterion to the fossil coral record.

## Material

Material used in this study consisted of skeletons of modern (i.e., alive until sampled) corals and one fossil specimen. Skeletons of modern corals selected from different museum collections represent zooxanthellate and azooxanthellate species (all taxonomic, locality and depth data are listed in S1 Table). Similar number of modern zooxanthellate (14 genera represented by 16 species) and azooxanthellate (13 genera represented by 13 species) taxa were selected for microstructural analyses. Selected corals represent all 3 major clades of Scleractinia: Basalia, Complexa, and Robusta that, except of solely azooxanthellate Basalia, include both zooxanthellate and azooxanthellate representatives. Extant zooxanthellate *Madracis decactis* (Lyman, 1859), *Mussismilia hispida* (Verrill, 1901) and azooxanthellate *Phyllangia americana* Milne-Edwards & Haime, 1849, and *Tubastraea tagusensis* Wells, 1982 were collected specifically for this study (authorized by Brazilian Environmental Ministry under the IBAMA permit #36717–1 (15-10-2012), CITES permit #13BR011219/DF) from a restricted area (ca. 5m^2^) at IIha dos Buzios island (Brazil). In the present study we also used remarkably well-preserved fossil skeleton of *Volzeia* sp., collected from lower Norian outcrops of the Lycian Taurus (near Gödene, Alakir Çay Valley, Antalya province, Turkey) [40]. All examined coral samples are housed at the Institute of Paleobiology, Polish Academy of Sciences, Warsaw (abbreviation ZPAL).

### Analytical techniques

Optical Microscopy [allows quick assessment of the organization of ultrastructural components of coral skeleton under transmitted-light]: Polished sections were examined using a Nikon Eclipse 80i transmitted light microscope fitted with a DS-5Mc cooled camera head. Observations were conducted in transmitted and polarized light at the Institute of Paleobiology, Warsaw, Poland.

Scanning Electron Microscopy (SEM) [provides high-resolution support of optical microscopy and allows obtaining more detailed information about textures of crystals in the thickening deposits]: Polished sections were lightly etched in Mutvei’s solution following described procedures [41], and then rinsed with Milli-Q water and air-dried. After drying, the specimens were put on stubs with double-sticking tape and sputter-coated with conductive platinum film. Analyses were made using a Phillips XL20 scanning electron microscope at the Institute of Paleobiology, Warsaw, Poland.

Transmission Electron Microscopy (TEM) [allows investigation of the nano- and microstructural characteristic of skeletal fibers as well as their crystallographic orientation]: Samples (ultra-thin foils) cut from the middle of the septa, were prepared using a FEI Helos Nanolab 600 dual beam machine equipped with Omiprobe nano-manipulator. Before FIB (focused ion beam) processing, the polished sections were covered by a 10nm thick platinum layer deposited by sputtering in a line across the region to be extracted. The selection of the exact area for FIB processing was performed by correlation with optical microscopy images. A 30kV Gallium ion beam was used to extract the skeleton lamella. Final thinning was performed on lamellae glued to the copper grid using 2KV acceleration voltage for the Ga^+^ ions. Transmission electron microscopy was performed at the Institute of Physics, Warsaw, Poland in STEM and TEM mode using FEI Titan 80–300 microscopes equipped with image corrector operating at 300 kV. For the STEM mode, the HAADF detector was used with a camera working distance of 360mm, which gives mixed diffraction like/chemical contrast.

Wavelength Dispersive Spectroscopy [used in order to determine spatial distribution of magnesium within fibrous part of the skeleton]: elemental X-ray mapping were acquired on a Cameca SX-100 electron microprobe at the Micro-area Analysis Laboratory (Polish Geological Institute, Warsaw) using described procedures [42]. The following conditions were used during stage scans: 15kV (accelerating voltage), beam current 5nA (for Calcium) or 20nA (for other elements), 60msec (pixel dwell-time), ca. 1μm raster step size, and 512 x 512 pixels. Specimens were coated with carbon of ca. 2nm thickness.

NanoSIMS [provides high-resolution spatial distribution of magnesium in thickening deposits of the coral skeleton]: The distribution of magnesium (^24^Mg/^44^Ca) was performed at the Laboratory for Biological Geochemistry (EPFL, Lausanne, Switzerland) on polished (0.25mm diamond suspension) and gold-coated (20nm) skeletal surfaces embedded in epoxy with the Cameca NanoSIMS 50L, following established procedures [43,44]. A primary beam of O^-^ (40–50pA) produced secondary ions of ^24^Mg^+^ and ^44^Ca^+^ that were transferred to the multi-collection mass-spectrometer and detected simultaneously in electron multipliers at a mass resolving power of ~5000. At this mass-resolving power, the measured secondary ions are resolved from potential interferences. First, spot-analyses were obtained from a pre-sputtered surfaces with the primary ions focused to a spot-size of ~800nm and the primary beam stepped across the sample surface with a step-size of 5 micrometers. The measured ^24^Mg/^44^Ca ratios were calibrated against analysis of a carbonate standard of known composition (OKA-C, [45]). The chemical variations recorded in the coral skeletons are much larger than both the internal and external reproducibility of the standard, which are typically less than 3% (2 standard deviations) for Mg/Ca in this analysis mode. Second, images of the Mg/Ca distributions were also obtained with the primary beam focused to about 450nm, typically on 40 x 40 mm^2^ surface areas with 256 x 256 pixels, and a pixel dwell-time of 5000 microseconds.

Cathodoluminescence Microscopy (CL) [a simple method used to determine spatial distribution of primary and secondary calcium carbonate polymorphs (aragonite and calcite respectively) within fossil coralla]: Cathodoluminescence of the skeleton of the Triassic *Volzeia* sp. was examined with a hot cathode microscope HC1-LM at the Institute of Paleobiology, Polish Academy of Sciences, operated with an electron energy of 14keV and a beam current density of 0.1μA mm^-2^.

Raman Microscopy [allows achieving high-resolution spatial distribution of calcium carbonate polymorphs within coral skeleton; verifies CL observations]: Raman maps of the Triassic *Volzeia* sp. skeleton were recorded at the Department of Chemistry, University of Warsaw, Poland with a LabRAM HR Raman confocal microscope (Horiba JobinYvon) equipped with a LPF Iridia edge filter, a 600 or 1800 groove mm^-1^ holographic grating and a 1024 x 256 pixel Peltier-cooled Synapse CCD detector. The microscope attachment was based on an Olympus BX41 system with an MPLN100x objective and a motorized, software-controlled x-y-z stage. The excitation source was the second harmonic of the diode-pumped Nd:YAG laser (Excelsior-532-100, Spectra-Physics) operating at 532.3nm with ca. 2mW power on the sample. The Raman spectra were recorded from selected area with 1μm x 1μm spatial resolution at 1s integration time. Polymorphs of calcium carbonate show various bands attributable to internal mode vibrations of the carbonate ion and rotational and translational lattice modes. For aragonite, the most intense peak appears at 1085cm^-1^, which is assigned to the symmetric stretching mode of the carbonate ion. The same band for calcite is only slightly shifted towards higher energy. A characteristic doublet assigned to the in-plane bending mode of CO_3_^2-^ anion is seen at ca. 701 and 705cm^-1^ in the spectrum of aragonite. These peaks are absent in calcite. Instead, a single band is observable at 711cm^-1^. The most convenient signals allowing identification of the polymorph are grouped in the 100cm^-1^–300cm^-1^ region. These peaks, associated with lattice vibrations, appear at 205cm^-1^ and 153cm^-1^ for aragonite. For calcite, the bands can be found at 281 cm^-1^ and 154 cm^-1^. The maps were extracted from raw Raman data through a direct classical least squares (DCLS) modeling procedure performed on the multidimensional spectral array. At each spectrum within the array the DCLS modeling procedure finds a linear combination of the reference component spectra that best fits the data. The reference spectra were selected from the raw data at specific positions where only aragonite or calcite were present, respectively.

**Fig 1:**
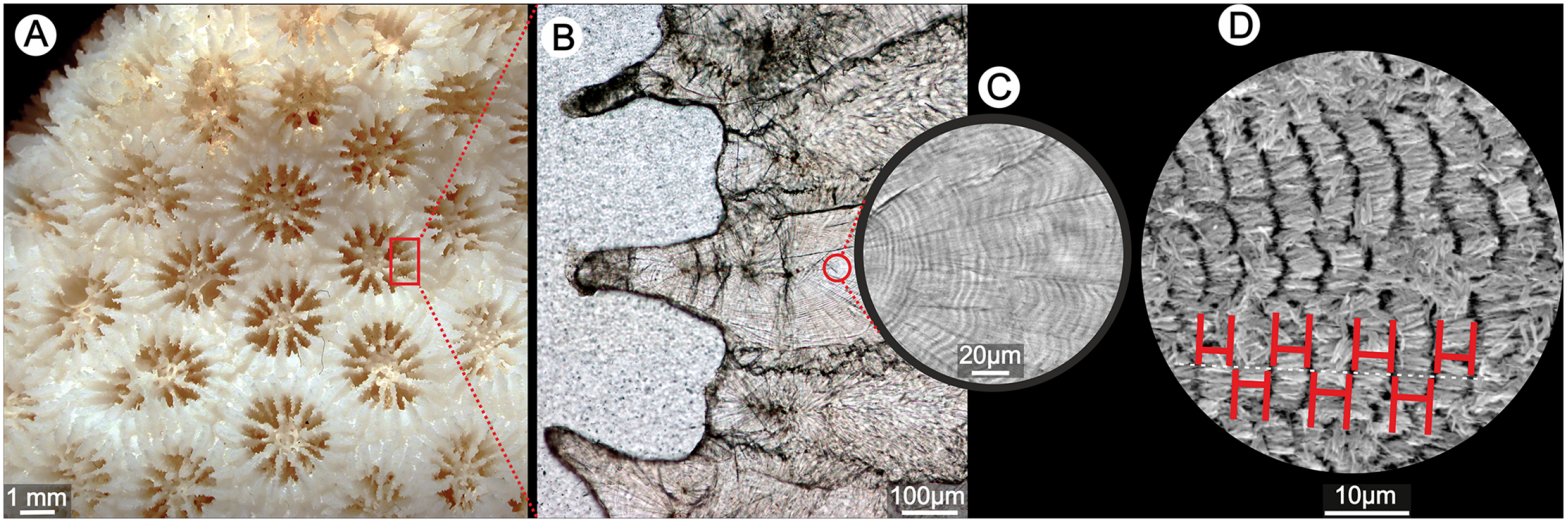
Macro- (A) and microscale (B-C) organization of extant scleractinian skeleton (*Goniastrea stelligera* ZPAL H.25/47) and methodology to measure the growth increments (D). Distal view of colony (A) from which a fragment (red rectangle) was thin-sectioned (B). Thickening deposits show doublets of optically light and dark bands (c, optical microscope). Thickness of growth bands (D, SEM after light etching) is measured along individual fibers (white-dashed line) and consistently at the beginning and end of each zone with positive etching relief (red segments).

Analysis of growth band regularity: Thickness of growth increments was measured along individual fibers on SEM photomicrographs of polished and etched surfaces of septa and walls (illustrated in Fig 1) for species of both modern zooxanthellate and azooxanthellate corals, listed in S1 Table. For each specimen the mean value (μ), standard deviation (σ) and coefficient of variation (CV) (i.e. ratio of the standard deviation σ to the mean μ; CV = σμ) of the measured growth band widths were obtained (20 measurements per distinct bundles of fibers). Regularity of growth bands was compared using the CV. The *t*-test was used to assess the differences in banding regularity between zooxanthellate and azooxanthellate groups–the null hypothesis is that there is no difference in regularity of micro-scale growth bands between these two ecological groups of corals. The results were considered as statistically significant at the 0.05 level. All statistical analyses were conducted using Past 3.07 software [46].

## Results

### Recent scleractinians

Coral skeletons are structurally characterized by two main components: 1) Rapid Accretion Deposits (RAD, traditionally referred to as Centers of Calcification), which represent about 2–5 volume % of the skeleton and in transverse thin sections are recognizable as optically dark spots or narrow, continuous regions [39,47,48,49]. 2) Thickening Deposits (TDs; traditionally referred to as “fibers”), representing the vast majority of the skeletal volume [39], and exhibiting fine-scale growth banding [46], which is best visible in sections made parallel to the growth axis of TDs, i.e., in transverse sections of skeletal structures (usually septa, but also massive walls). First, in order to study the structural and chemical features of growth banding, a number of corals with particularly well developed banding were characterized using an array of microscopic imaging techniques.

#### Structure and geochemistry of growth bands

**Fig 2:**
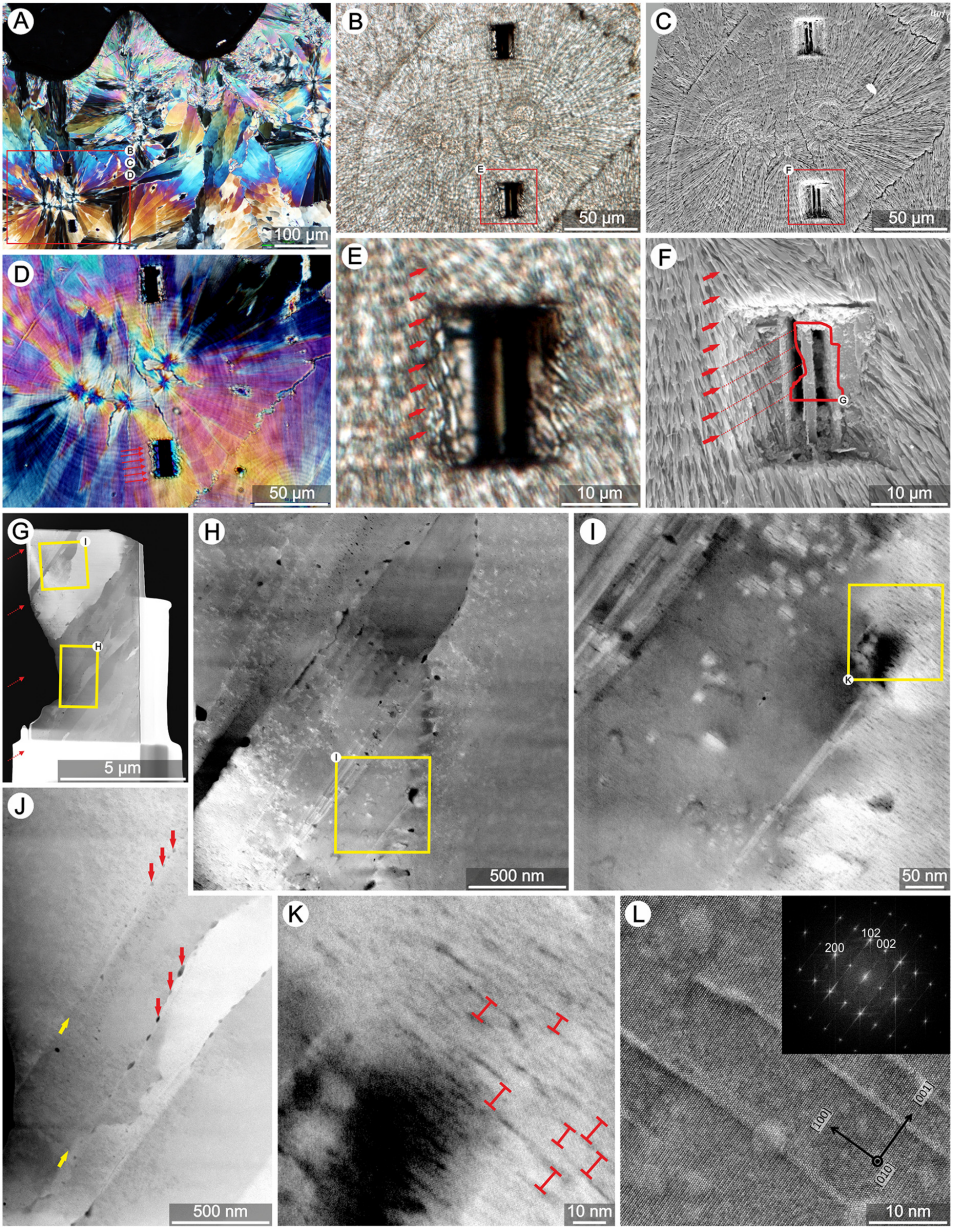
Structure of banded TDs in skeleton of extant zooxanthellate coral *Goniastrea stelligera* (ZPAL H.25/47). Transmitted light microscopy (polarized **A**, normal light **B, E**, and Nomarski Interference Contrast **D**), and corresponding Scanning Electron Microscopy images (SEM; polished and etched section, **C**, **F**). Fibers within individual bundles have ordered crystallographic structure producing complete light extinction every 90° during rotation of the section in polarization microscope. Darker layers within Thickening Deposits (TD) in transmitted light (e, red arrows) correspond to regions with negative etching relief (F). Conversely, light layers show positive etching relief. There are ca. 6 bands (E–F) in the region from which FIB thin section was extracted (about 3 that should occur in the TEM examined section, G). TEM bright field image of FIB lamella (**G**) with few relatively large monocrystalline fibers (contrast related to different orientation of fibers); yellow frames mark enlarged areas (**H**, **I**, **J**, **K**). On the fibers boundaries (yellow arrows in J) occur voids (ca. 10–65nm in length, red arrows in J) that most likely represent organic material inclusions trapped between growing fibers. Enlarged regions of fibers (I, K, L) show numerous nano-voids (ca. 10–40 nm long and 1–2 nm wide), which are perpendicular to *c*-axis of aragonite fibers. Distance between nano-voids in fiber's *c*-axis direction is ca. 10nm (e.g., red bars in K). All diffraction peaks on FFT of image (insert in L image) where indexed within the orthorhombic symmetry system (space group Pmcn (62)) with lattice parameters a = 4.9623Å, b = 7.968Å, c = 5.7439Å.

**Fig 3:**
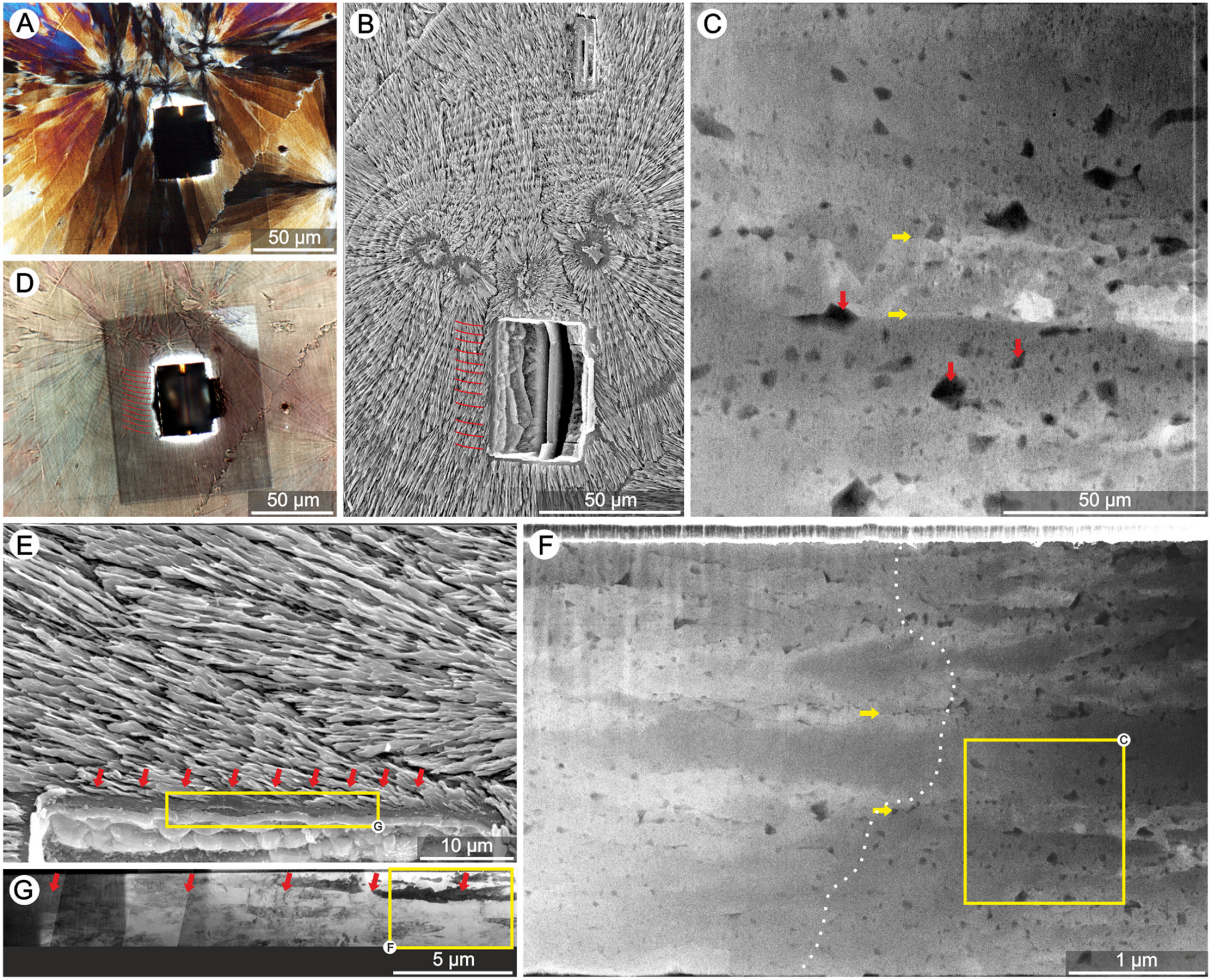
Structure of banded Thickening Deposits in skeleton of extant zooxanthellate coral *Goniastrea stelligera* (ZPAL H.25/47). Transmitted light microscopy (polarized **A**, normal light **D**, and corresponding Scanning Electron Microscopy images (SEM; polished and etched section, **B, E**). TEM bright field (BF) images (**C**, **F**, **G**) of 21.5μm long FIB lamella (yellow rectangle marks approximate position on etched polished section near border of “FIB crater”). Composite image of FIB lamella (G) consists of aligned of 7 individual images. The contrast difference is related to variation of the lamella thickness; image contrast and clarity of image drops with the lamella thickness. Orientation of FIB lamella is approximately parallel to *c*-axis of aragonite fibers which can be distinguished due to differences in orientation (boundaries of some fibers marked with yellow arrows in C, F); length of most of fibers exceeds 10 μm which is consistent with SEM images. STEM-HAADF image (F) of the thinnest part of lamella (frame in g); camera length was fixed to 360mm and contrast is related to elastically scattered electrons i.e., orientation of the crystals and average local thickness. The black dots correspond to the voids into structure (C, enlarged part of F). Density of voids is not homogenous—dotted-line in F was subjectively drawn to distinguish zone with particularly numerous voids and zone with relatively few voids. Voids (especially in region right to the dotted line) are also present in monocrystalline fibers suggesting encapsulation of organic material during fiber grow. Region rich in voids most likely corresponds to darker zones in optical microscope: light scattering by voids and by smaller-size crystals differs from that of long monocrystalline fibers. Also etching of defective zone is faster that may explain differences in etching relief of darker vs. lighter banding.

Skeletons of several coral species were selected to obtain in-depth characterization of TDs. The skeleton of zooxanthellate coral *Goniastrea stelligera* shows simple organization of TDs (Fig 2): large bundles of fibers are oriented perpendicular to the skeletal surface. Fibers within individual bundles have ordered crystallographic structure (Figs 2A, 2D and 3A) [50]. Regular growth increments are usually 3–6μm wide, each consisting of a doublet of optically light and dark bands assumed to represent daily growth increments [51,52,53]. Micro-scale bands are usually easy recognizable in normal transmitted light and Nomarski Interference contrast improves their detection in polarized light (Fig 2D). SEM micrographs of lightly etched skeletal samples show regular etching relief with intercalating negatively and positively etched layers. Layers darker in transmitted light correspond to regions with negative etching relief (Fig 2B, 2C, 2E and 2F). Conversely, optically light layers show positive etching relief. Progressively etched surfaces show also that fibers are generally continuous between growth layers, but appear to narrow and eventually become discontinuous with prolonged etching of dark layers (Fig 3E).

To visualize smaller-scale structure of the growth bands, several FIB thin-sections were extracted from the banded regions of thickening deposits (Figs 2 and 3), containing 2 to 5 growth bands (see Figs 2E–2G, 3B, 3E and 3G). At first glance, TEM bright field images of FIB lamellae do not show any clear banding pattern within fibers. Fibers consist of relatively large monocrystals that, due to slightly different crystallographic orientation (a-b axes), exhibit contrast differences in bright field TEM images (e.g., Figs 2G, 2J, 3F and 3G).

At higher magnification, several voids (ca. 10–65nm in length) are visible, especially at fiber boundaries (Fig 2J, yellow arrows). Enlarged images of fibers (Fig 2I, 2K and 2L) also reveal much smaller and numerous voids (ca. 10–40nm long, 1–2nm wide, and separated from each other by ca. 10nm in the *c*-axis direction) that are oriented perpendicular to the *c*-axis of aragonite fibers. A heterogeneous distribution of micro-scale voids could be observed in the thinnest part of longer FIB lamella (Fig 3G). In this region, a zone with numerous voids (Fig 3F, right to the dotted-line) and a zone with rare voids (left to the dotted line) were observed. The region with numerous voids seems to correspond to the negatively etched zone in SEM, although the boundary between these two structural regions can be difficult to distinguish.

**Fig 4:**
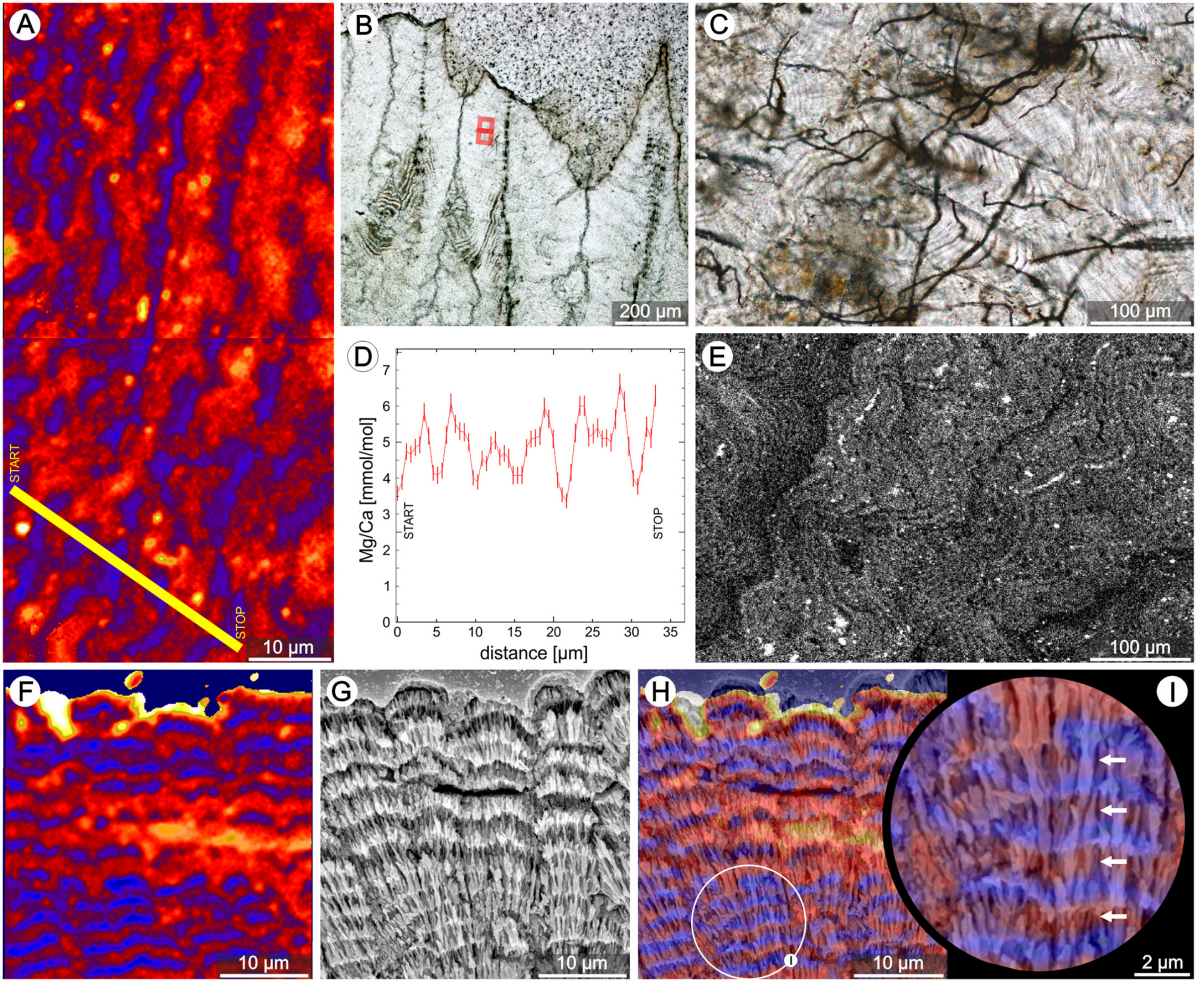
Some geochemical and structural features of regularly banded TDs of extant zooxanthellate corals. (**A**) The distribution of magnesium (^24^Mg/^44^Ca) in TDs of *L*. *scutaria* (ZPAL H.25/49); NanoSIMS image—mapped regions marked with red rectangles in **B** (thin-section in transmitted light). Blue colors correspond to relatively low Mg concentrations; red to yellow colors correspond to increasingly high Mg concentrations. (d) NanoSIMS profile of Mg/Ca across banded region of TDs (profile region marked with yellow line in A). (C,E) Transverse section of the skeleton of *C*. *lacrymalis* (ZPAL H.25/43) in transmitted light (C), and mapped with Wavelength Dispersive Spectroscopy technique (E) to show distribution of Mg. Consistent with the NanoSIMS maps, layers of the fibrous skeleton (TDs) exhibit micro-scale zoning in Mg concentration. The distribution of Mg in *P*. *damicornis* (ZPAL H.25/54) skeleton visualized with NanoSIMS (F), the corresponding skeletal ultrastructure (g, etched polished section of the region imaged in F), and the two images superimposed (H). Enlarged part of H (I, encircled) suggests that regions showing negative etching relief are enriched in Mg (white arrows in I).

In addition to structural banding, a distinct micro-scale zonation in Mg-distribution (^24^Mg/^44^Ca) was observed, e.g., in skeletons of zooxanthellate *Lobactis scutaria* and *Pocillopora damicornis* (Fig 4A and 4F, NanoSIMS mapping) and *Cynarina lacrymalis* (Fig 4E, WDS mapping). The layered structure of TDs in *P*. *damicornis* is well organized (Fig 4G) allowing precise correlation of structural and geochemical images: regions with relatively high Mg concentration correspond to negative etching relief (etching performed after NanoSIMS analysis), whereas regions with positive etching relief are relatively low in Mg concentration (Fig 4F–4I).

#### Growth band regularity in zooxanthellate and azooxanthellate corals

**Fig 5:**
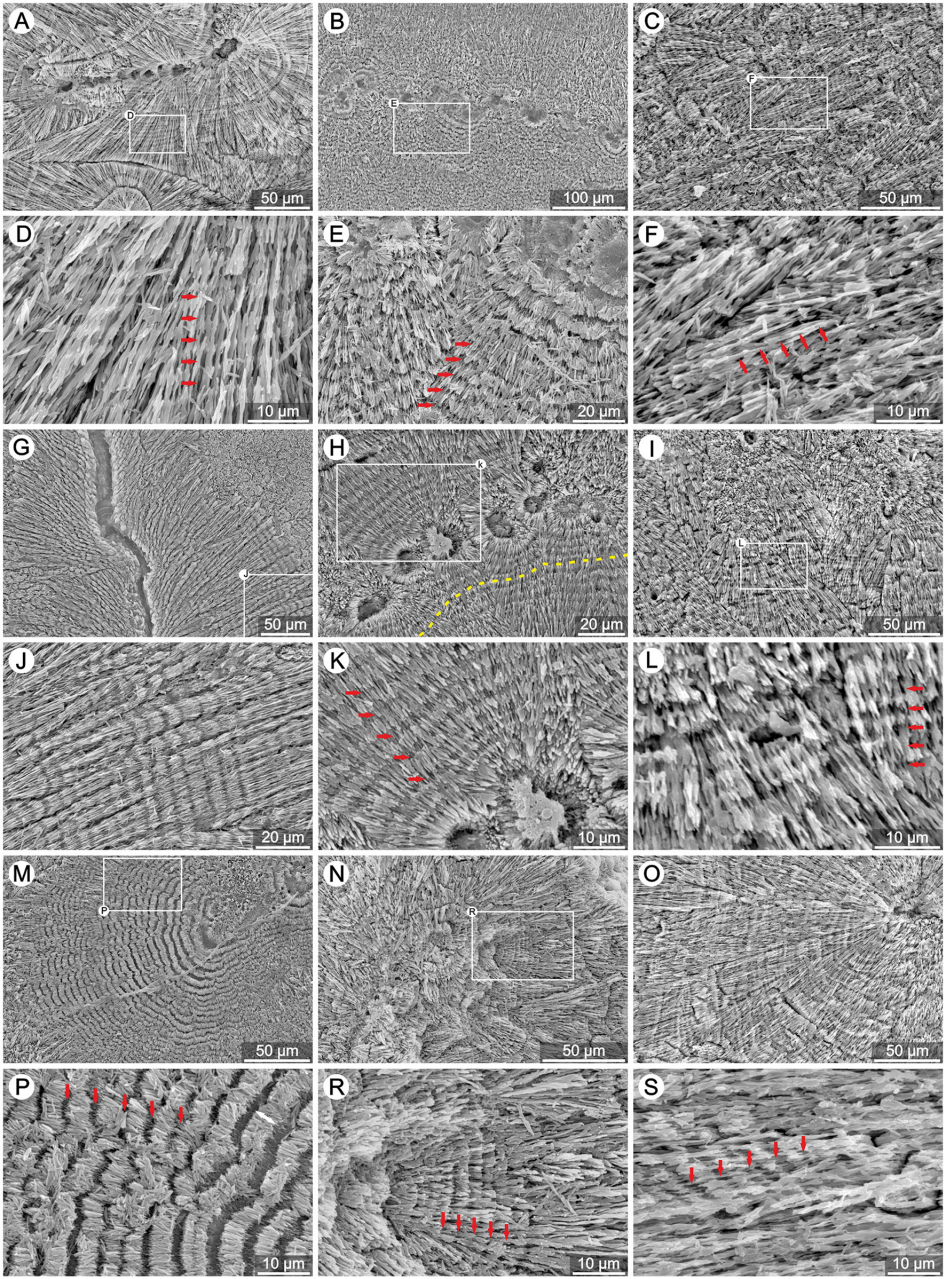
Regular banding of TDs in extant zooxanthellate corals. SEM micrographs of polished and etched skeletons of various zooxanthellate corals (photographs are in pairs: upper and lower photo): (A,D) *Goniastrea stelligera* (ZPAL H.25/47), (B,E) *Symphyllia valenciennesii* (ZPAL H.25/57), (C,F) *Lobophyllia hemprichii* (ZPAL H.25/50), (G,J) *Pocillopora damicornis* (ZPAL H.25/54), (H,K) *Porites porites* (ZPAL H.25/55), (I,L) *Goniastrea retiformis* (ZPAL H.25/45), (M,P) *Acanthastrea echinata* (ZPAL H.25/42), (N,R) *Galaxea fascicularis* (ZPAL H.25/44) and (O,S) *Merulina ampliata* (ZPAL H.25/52). Thickening Deposits form regular (red arrows) and continuous layers (e.g., yellow dotted line in H).

**Fig 6:**
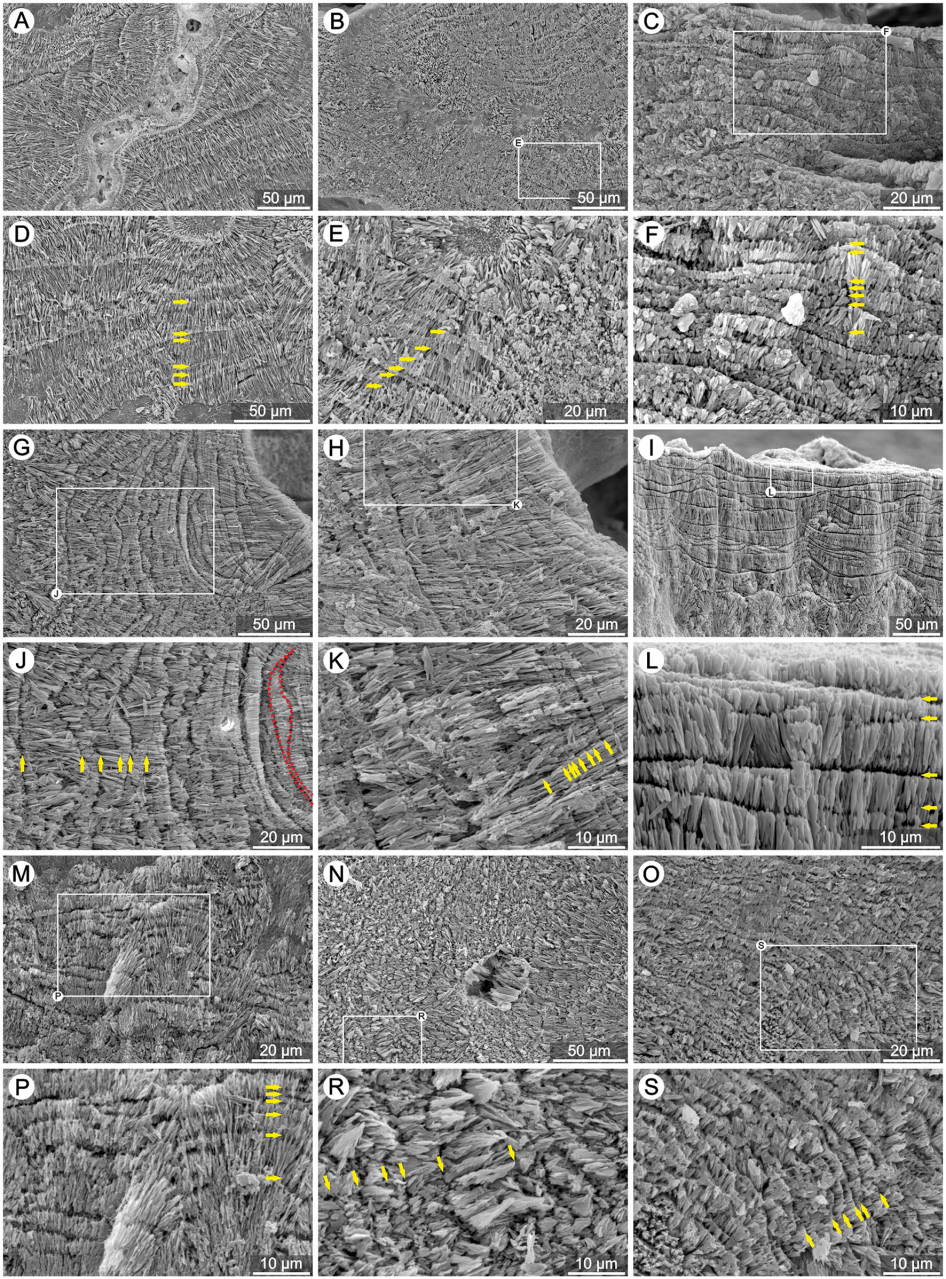
Growth increments of TDs in extant azooxanthellate corals. SEM micrographs of polished and etched skeletons of various azooxanthellate corals (photographs are in pairs: upper and lower photo): (A,D) *Bathelia candida* (ZPAL H.25/59), (B,E) *Cyathelia axillaris* (ZPAL H.25/61), (C,F) *Hoplangia durotrix* (ZPAL H.25/65), (G,J) *Caryophyllia inornata* (ZPAL H.25/60), (H,K) *Paracyathus pulchellus* (ZPAL H.25/68), (I,L) *Desmophyllum dianthus* (ZPAL H.25/62), (M,P) *Stephanocyathus paliferus* (ZPAL H.25/70), (N,R) *Leptopsammia pruvoti* (ZPAL H.25/66) and (O,S) *Gardineria* sp. (ZPAL H.25/64). Thickening Deposits form irregular (yellow arrows) and discontinuous layers (e.g., red dotted line in J).

All coral samples examined in this study exhibited the typical micro-scale organization of the skeleton with central regions of septa consisting of RAD (examples Figs 5A, 5B, 5G, 5H and 6A) and the main skeletal mass formed by TDs. At glance, TDs of zooxanthellate corals showed regular banding (typical spacing ca. 4–6μm; Fig 5) whereas such patterns were not obvious in azooxanthellate corals (Fig 6). In zooxanthellates, individual layers usually have similar thickness (e.g., *S*. *valenciennesii* in Fig 5B and 5E), growth layers are continuous over larger portion of TDs (e.g., *P*. *porites* (Fig 5H, yellow dotted line). Less regular incremental bands in symbiotic corals *L*. *fragilis* and *M*. *decactis* (S2A and S1B Figs) are exceptions.

Conversely, and by direct comparison, banding in the vast majority of the studied azooxanthellate coral skeletons is clearly irregular (Fig 6). Differences in thickness occur between adjacent layers as well as within single bands, with thickness ranges from ca. 1μm (e.g., *P*. *pulchellus* in Fig 6K) to several/dozens of μm (e.g., *L*. *pruvoti* in Fig 6R). The layers are often discontinuous, illustrated in the skeleton of *C*. *inornata* (red dotted line in Fig 6J) and *P*. *americana* (white dotted line in S2C Fig). As the only exception, the TD banding of azooxanthellate *T*. *tagusensis* is regular and continuous over larger portions of the skeleton (S2D Fig).

### Triassic scleractinian

**Fig 7:**
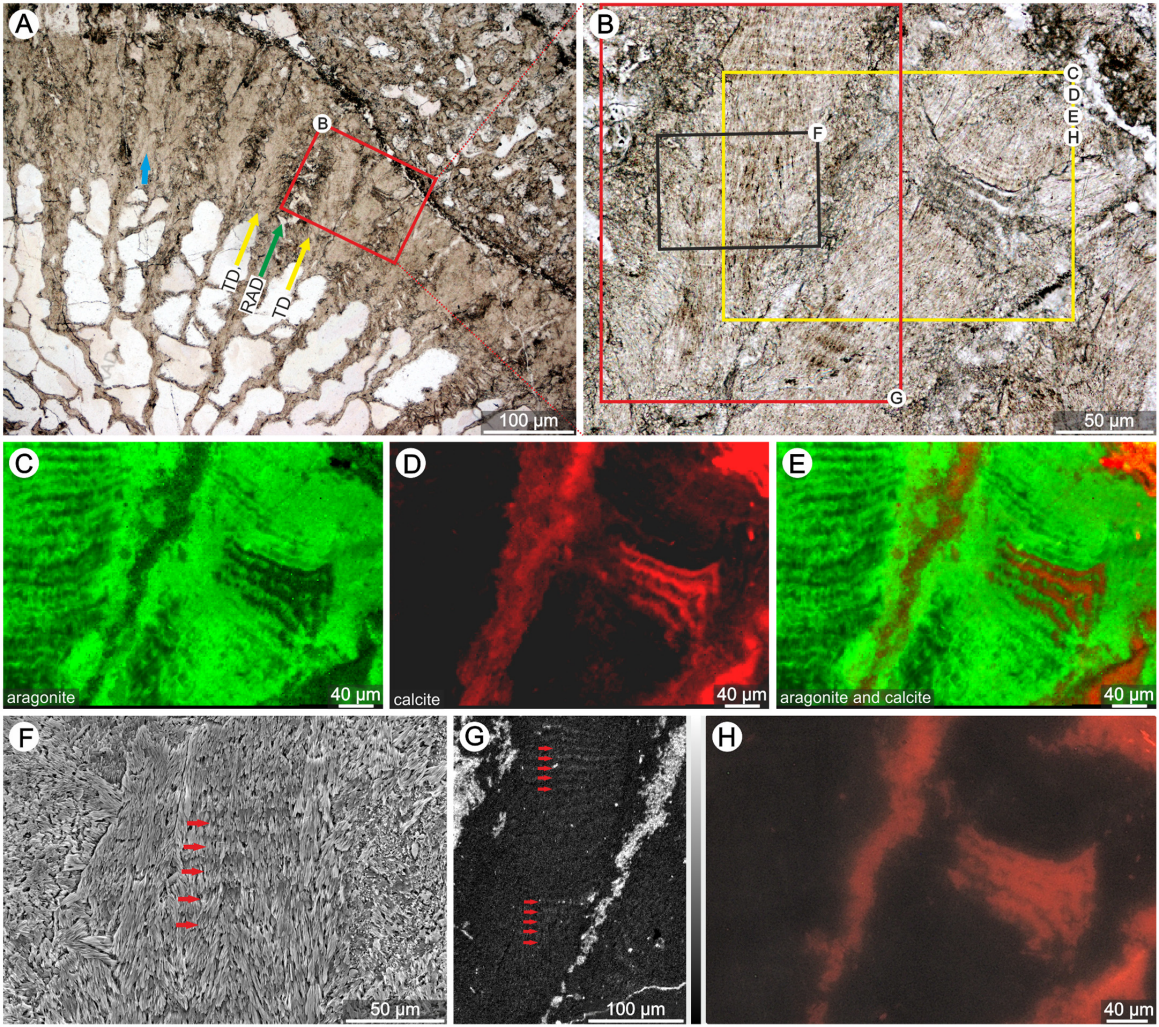
Regular growth increments of TDs and some mineralogical features of the skeleton of the Triassic (Lower Norian) *Volzeia* sp. from Alakir Ҫay, Turkey (ZPAL H.21/26). Optical microscope images of transverse thin sections of the skeleton (A,B). Yellow rectangle in B indicates area selected for micro-Raman (C-E) and cathodoluminesence microscope (H), black rectangle in B indicates area selected for SEM (F), and red rectangle in B indicates area selected for WDS analysis (G). Regions of TDs with regular growth increments (enlarged in B) may co-occur with those without banding (blue arrow in A). Micro-Raman maps of aragonite (C), calcite (D) and superimposed images of both polymorphs (E) extracted from raw Raman data through a direct classical least squares (DCLS) modelling procedure. SEM micrograph (F) shows presence of regular growth layers (red arrows). WDS map (G) shows distribution of Mg. White color corresponds to high Mg concentrations; black color corresponds to low Mg concentration. Red luminescence on CL image (H) corresponds to regions composed of calcite, whereas the absence of luminescence corresponds to regions composed of aragonite.

Transverse thin-sections of the skeleton of the phaceloid *Volzeia* sp. show well-preserved microstructural details: RADs (green arrow) and TDs (yellow arrow; Fig 7A). Close-up of the corallite wall reveals partially preserved banding pattern (Fig 7B), which is the first case of distinct micro-scale banding within TD deposits illustrated in Norian (Triassic) corals [33]. Regions of TDs within the same thin-section, but without banding, are also observed (blue arrow in Fig 7A). Secondary calcite (green color in Raman map and red luminescence in CL image; Fig 7D, 7E, 7H, respectively) fills the RADs structure. Fibrous parts of the skeleton are composed predominantly of aragonite (blue on Raman map and black in CL image), and only small regions within TDs are partly calcitic–calcite appears as alterations between aragonite crystals (Fig 7C–7E and 7H). High Mg content is observable in RADs zones as secondary calcite infilling, in secondary calcite alterations between aragonite crystals, but also–with lower concentrations and similar to extant corals–as regular Mg banding within aragonite fibers (Fig 7G). Regular growth layers, similar to those observed in transmitted light, are visible in lightly etched sections in SEM (Fig 7F). Individual layers have similar thickness and are continuous over larger portion of TDs: their mean thickness value is 10μm and standard deviation is 1.14μm; all measurements were made in regions of the *Volzeia* sp. skeleton that showed banding in original aragonite mineralogy.

### Statistical analysis of growth increments

**Fig 8:**
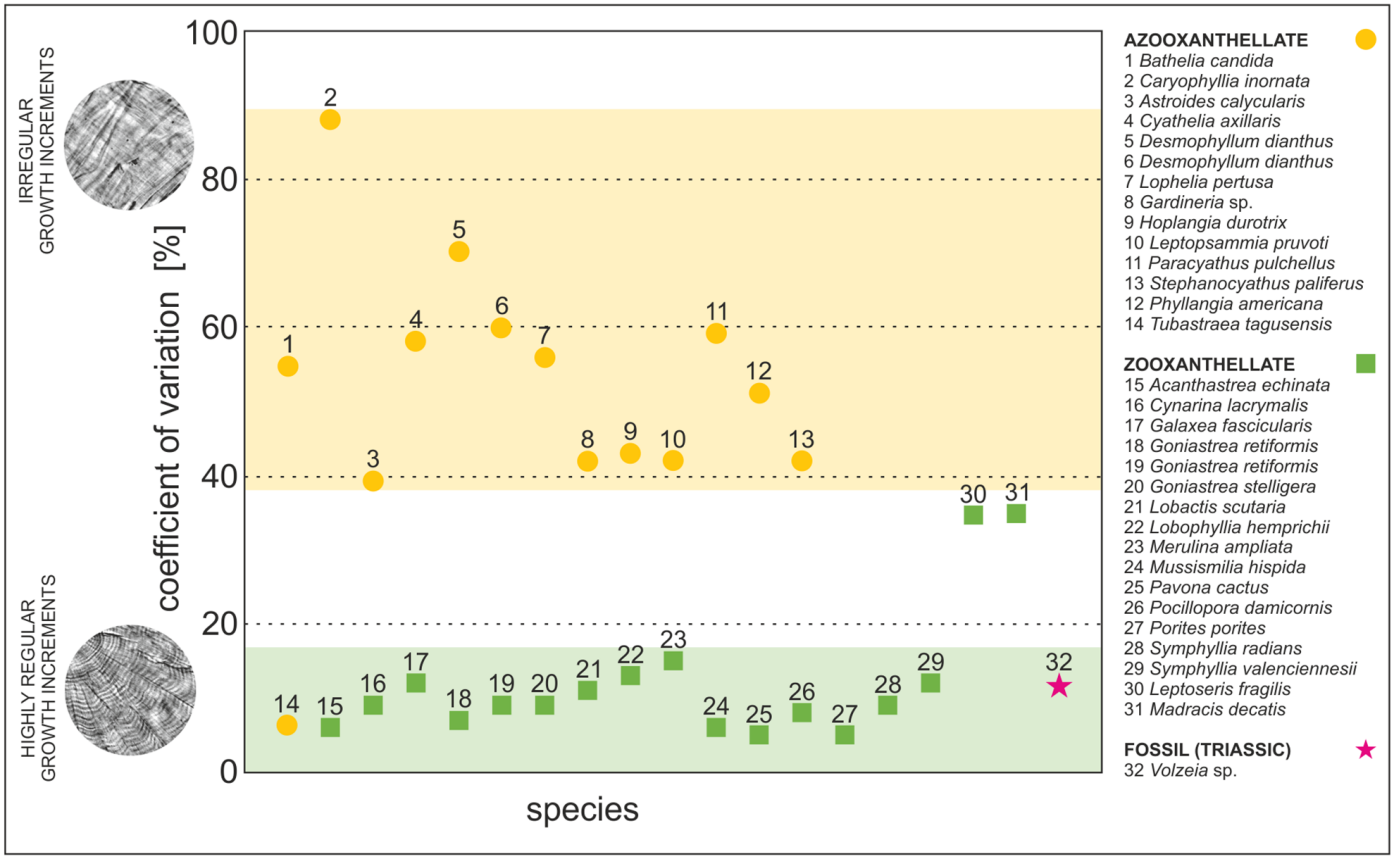
Statistical analysis of regularity of growth increments in modern and fossil Scleratinia. Plot illustrates relationship between regularity of increments (expressed as coefficient of variation [%] of dispersion of values of bands thickness obtained from each skeleton) and presence/lack of endosymbiotic algae. Thickness of growth bands was measured along individual fibers (red marks in image in the upper-right corner). Orange dots indicate azooxanthellate corals, green squares are zooxanthellate corals, pink star is exceptionally well preserved fossil (Triassic) specimen from Antalya, Turkey. Database of all measurements is given in S1 Table.

We used the coefficient of variation (CV) as a simple and easily obtainable measure of dispersion of band thickness variations among all studied corals (Fig 8, S1 Table). With only one exception (out of 12), the CV for azooxanthellate corals ranges from ~40 to ~90% and indicates moderate to high irregularity within examined skeletal regions. On the contrary, the vast majority of examined zooxanthellate species (with only two exceptions out of 16) are characterized by low value of CV (~5 to ~15%), indicating that their growth increments are very- to highly regular. When compared, there is significant difference of regularity between symbiotic and asymbiotic groups of corals (*t* test, p<0.001). Nonetheless, some departures from this general rule are observed in both ecological groups. Azooxanthellate *T*. *tagusensis* skeleton has a CV about 6% and based solely on this parameter could be wrongly assigned as symbiotic coral. The CVs obtained for two zooxanthellate corals, *L*. *fragilis* and *M*. *decactis*, are about 35% and would thus (wrongly) suggest azooxanthellate character of these corals. Despite of these few exceptions, the banding pattern in great majority (>90%) of examined corals differs distinctly between zooxanthellate (CV<20%) and azooxanthellate corals (CV>40%). Coefficient of variation for the Triassic *Volzeia* sp. is about 11% that is consistent with regular banding of modern zooxanthellate corals (Fig 8, pink star).

## Discussion

### Structural and geochemical characteristics of growth bands

Our observations confirm that fibers in extant scleractinian corals are aragonitic monocrystals (often several tens of micrometers in length, e.g., Fig 3F and 3G) that pass without crystallographic disruption across regions corresponding to fine-scale optical banding [50,51,52,53]. However, at higher magnification these fibers reveal zones with numerous voids, which correspond to the negatively etched regions in SEM, and zones with rare voids, which correspond to regions with positive etching relief in SEM (Fig 3F). Moreover, micro-scale Mg (Fig 4) [43] and S [54] zonation within fibers was shown to have the same periodicity as structural banding. Correlating SEM structural and NanoSIMS Mg distribution images clearly shows that regions exhibiting negative etching relief are enriched in Mg, whereas regions with positive etching relief are relatively Mg-poor (Fig 4F–4I). This observation is consistent with work by Motai et al. [55] who suggested that differences in elemental concentration are responsible for different response to etching (different resistance to ion sputtering).

To date, there is no commonly accepted model of Mg incorporation in aragonite lattice in scleractinian corals and it is unclear whether the carrier of Mg is organic phase or highly disordered mineral phase. Finch & Allison [56] suggested that skeletal Mg can be organically complexed and the voids within aragonite fibers documented in this study may represent such originally Mg-rich organic phase inclusions. This interpretation may also explain the preferential etching of Mg-enriched zones because the presence of voids increases the surface area and the rate of dissolution. Such preferential etching occurs also along the borders of individual fibers, where larger voids are present, conceivable also representing organic material trapped between growing fibers (Fig 2J). In addition to these ‘larger’ voids (about 100nm in size) we also observed occurrence of much smaller nano-voids (ca. 10–40nm long, 1–2nm wide) regularly separated from each other by ca. 10nm along the aragonite fiber *c*-axis (Fig 2I, 2K and 2L). These nano-voids may represent intra-crystalline inclusions of organic macromolecules that are directly involved in biomineralization process and which might be responsible for systematic deviations of the lattice parameters of biogenic aragonite with respect to reference (non-biotic) aragonite [57,58]. These observations suggest that biological modulation of the biomineralization process occurs at various length scales with rather smooth spatial transitions that contribute to micro-scale skeletal phenomena.

#### Microstructural criterion of zooxanthellate symbiosis in modern and fossil scleractinian corals

Observations made in this paper and in the literature [39,50,55,59] suggest that regular vs. irregular growth increments of thickening deposits in scleractinian corals can be linked with the presence or absence of symbiotic algae. Two distinct groups of corals are distinguished based on differences in regularity of growth banding (Fig 8; S1 Table): The first group, characterized by low values of CV: ~5 to ~15% (regular banding) consists almost exclusively of zooxanthellate corals (green zone in Fig 8); the second group with high values of CV: ~40 to ~90% (irregular banding) is formed primarily by azooxanthellate corals (yellow zone in Fig 8).

Several factors directly linked with presence of zooxanthellae may modulate calcification in symbiotic corals in a diurnal manner resulting in regular day/night banding (S3 Fig). Among them, the light-dependent circadian regulation of carbonic anhydrase (CA) genes expression [60] may play an important role. The CA in zooxanthellate corals provides high amounts of inorganic carbon to their intracellular symbionts stimulating their activity and also converts metabolic CO_2_ into the bicarbonate anion [61,62]. Other factors that may enhance daytime calcification and which are directly related to photosymbiotic activity include: (i) increased supply of photosynthetic oxygen [62,63,64] or (ii) H_2_O_2_ [52,65,66], (iii) enhanced assimilation by the zooxanthellae of phosphates (coral-metabolism derivatives) [67,68], which are considered as inhibitors of calcification [69], (iv) photosynthesis-induced secretion of hydroxyl ions [70] enhancing diffusion of H^+^ ions from the calcification site and thus calcification, and (v) organic matrix synthesis and the supply of precursor molecules as a result of photosynthetic activity [71].

Although the all above mentioned factors may contribute to the presence of regular banding in skeleton of the majority of zooxanthellate corals (and lack of such regularity in majority of azooxanthellate corals), the exceptions identified in this study (i.e., quite regular banding in azooxanthellate shallow-water *Tubastraea tagusiensis* and relatively irregular banding in two zooxanthellates: deep-water *Leptoseris fragilis* and shallow-water *Madracis decactis*) require alternative explanation(s). A regular growth rhythm seems imposed on calcification process of *Tubastraea tagusiensis* and, conversely, the normal diurnal calcification dynamics of two zooxanthellate corals seem to be down-regulated.

Heterotrophic feeding was shown to have positive influence on coral biomineralization [72,73]. Thus, feeding at irregular or regular intervals may drive the irregular (or conversely, in exceptional cases, highly regular) banding of azooxanthellate corals. Similarly, the observed lack of regular growth increments in deep-water zooxanthellate *L*. *fragilis* may result from the combination of irregular heterotrophic, filtratory feeding [74] and a weak diurnal cycle due to the depth of its habitat. Experimental studies on zooxanthellate and azooxanthellate coral physiology and feeding behavior may provide further understanding of the factors that control the growth banding. The recently described night-time formation of thickening deposits [75] might represent another factor that could contribute to the irregularity of the skeletal growth bands.

Although our microstructural criterion shows some limitation, it does hold great potential for distinguishing zooxanthellate and azooxanthellate corals, including cases where the traditional morphological criterion [21] fails (e.g., the solitary but zooxanthellate coral *C*. *lacrymalis* shows regular micro-scale bands, whereas colonial but azooxanhtllate coral *A*. *calycularis* have irregular increments; S1 Fig). The exceptionally well-preserved Triassic *Volzeia* sp. skeleton studied here (Fig 7C–7E and 7H) has thickening deposits composed predominantly of aragonite fibers (confirmed by Raman maps as well as CL images—diagenetic calcite appears mainly as infilling of RAD areas, Fig 7E and 7H). The coral has a phaceloid low-integrated growth form but shows regular micro-scale bands of TDs (CV of 11.45%), which is similar to the regular banding of modern zooxanthellate corals. Additionally, some micro-scale bands are partially, neomorphically recrystallized resulting in a fine-scale regular banding very similar to the Pleistocene *Orbicella annularis* [76], a species that today is exclusively symbiotic.

## Conclusions

- Growth of the fibrous part of the skeleton (Thickening Deposits) of scleractinian corals happens in growth increments composed of light and dark doublets. Combined structural (SEM, TEM) and geochemical (NanoSIMS) analyses revealed that optically dark bands are enriched in Mg and contain numerous nano-scale inclusions, most likely of organic macromolecules, and exhibit negative etching relief. Optically light layers are characterized by a distinctly lower content of Mg, fewer inclusions and, consequently, have positive etching relief.
- Micro-scale growth increments in zooxanthellate corals are highly regular and continuous, whereas in corals without symbionts the increments are irregular and often discontinuous. Regularity of growth bands (expressed as coefficient of variation) ranges from ~40 to ~90% in azooxanthellate corals and from ~5 to ~15% in symbiotic species.
- Despite a few observed exceptions (3 corals out of 38 studied), the microstructural criterion expressed as a simple coefficient of variation appears to be a robust tool for discriminating between zooxanthellate and azooxanthellate coral forms. From the fossil record, we report the first regular fine-scale banding of thickening deposits in a Triassic scleractinian (*Volzeia* sp.) suggesting that this coral was symbiotic with dinoflagellates.

The main conclusion of this work is that microstructural features should be included with other proxies to study symbiosis in fossil corals.

## Supporting Information

S1 Fig Growth increments of TDs in some zooxanthellate (A,B) and azooxanthellate (C,D) corals whose morphological or microstructural features do not fit traditional criteria of symbiotic vs. non-symbiotic relationships. Microstructure of morphological “exceptions”: large and solitary zooxanthellate *Cynarina lacrymalis* (ZPAL H.25/43) (A) forms regular growth increments, whereas colonial and azooxanthellate *Astroides calycularis* (ZPAL H.25/58) (C) forms irregular bands. Exceptions from microstructural criterion: growth increments in skeleton of deep-water symbiotic coral *Leptoseris fragilis* (ZPAL H.25/48) (B) is not as regular as in many shallow-water zooxanthellate taxa, whereas in shallow-water (4–6m depth) asymbiotic *Tubatraea tagusensis* (ZPAL H.25/71) (D) banding pattern is clearly regular. Red arrows = regular bands, yellow arrows = irregular bands. SEM micrographs.
(PDF)

S2 Fig Growth increments of TDs in zooxanthellate (A,B) and azooxanthellate (C,D) corals collected from the same site (IIha dos Buzios, Brazil). SEM photomicrographs of modern zooxanthellate species: (A) *Madracis decactis* (ZPAL H.25/52) and (B) *Mussismilia hispida* (ZPAL H.25/53) and azooxanthellate species: (C) *Phyllangia americana* (ZPAL H.25/69) and (D) *Tubastraea tagusensis* (ZPAL H.25/71). Regular growth increments are observed in zooxanthellate *Mussismilia* and azooxanthellate *Tubastraea*, and irregular growth increments in zooxanthellate *Madracis* and azooxanthellate *Phyllangia*. Skeletal characteristics of these corals, which differ from the typically pattern of banding (i.e. regular in zooxanthellate and irregular azooxanthellate corals), most likely reflects physiological peculiarities of *Madracis* (slow growth rate) and *Tubastraea* (relatively fast growth). Red arrows = regular bands, yellow arrows = irregular bands.
(PDF)

S3 Fig Factors that potentially may induce day (A–F)–night (A’–F’) differences in scleractinian coral biomineralization. (A,A') Carbonic anhydrase. Expression of carbonic anhydrase is light-induced and its increased level during a day leads to increase of CO_2_ dehydration rate. High amount of bicarbonate ions delivered into calcification site together with increased activity of Ca^2+^-ATPase pump, results in enhanced skeleton growth. Conversely, low levels of this enzyme during the night restrict supply of HCO_3_^-^, that results in slower skeleton growth. (B,B’) Photosynthetic oxygen. During the day, oxygen released by photosymbionts is transported into calicoblasts where it enhances respiratory production of CO_2_ (later dehydrated by CA to bicarbonate ions) and ATP (used as calcium ion pump). High production of CO_2_ and ATP increases delivery of Ca^2+^ and HCO_3_^-^ ions to calcification site and results in higher rate of calcification. At night, when photosynthetic O_2_ delivery is inhibited, respiratory production of CO_2_ and ATP is low. Consequently, this limits supply of Ca^2+^ and HCO_3_^-^ ions and leads to decrease in growth rate. (C,C’) Photosynthetic hydrogen peroxide. H_2_O_2_ produced by zooxanthellae during photosynthesis cause lipid peroxidation of plasma membrane of calicoblasts thereby increasing leakage of Ca^2+^ ions into these cells. High concentration of calcium ions in calicoblasts results in their enhanced delivery into calcification site and higher growth rate. At night, when peroxide is not released, less Ca^2+^ ions is delivered to calicoblasts and calcification site, thus growth rate decrease. (D,D’) Metabolic phosphates. Phosphates produced in coral metabolism are regarded as inhibitors of skeletal growth. Their uptake by algae during photosynthesis cause that calcification process is not disturbed. When phosphates are not removed from the coral tissue during the night, they might reach calcification site and negatively affect deposition of carbonate. (E,E’) Hydroxyl ions. Photosynthesis-inducted secretion of hydroxyl ions in the coelenteron results in its alkalization and may facilitate diffusion of protons from calcification site. Removal of H+ ions from calcification site may in turn lead to enhancement of skeletal growth. When photosynthesis is inhibited, low pH in the coelenteron inhibits protons diffusion resulting in drop of calcification rate. (F,F’) Organic compounds. Zooxanthellae supply calicoblasts with organic matrix precursors (amino acids and glycerol), thereby increasing efficiency of skeleton formation during the day. During the night, when photosynthates delivery stops, secretion of OM and skeletal deposition decreases.
(PDF)

S1 Table Studied specimens data. Inventory (1st column) and repository (2nd column) numbers, taxonomic attribution (3rd column) and available locality data (4, 5 columns including [year of collection] and {position on attached map}) of examined modern coral samples. The 6th column shows zooxanthellate (z) or azooxanthellate (az) status of the coral. Next columns include information about mean values (7th column), standard deviation (8th column) and coefficient of variation (9th column) of measured growth bands.
(PDF)
