# Supplementary material for: Fine-Scale Skeletal Banding Can Distinguish Symbiotic from Asymbiotic Species among Modern and Fossil Scleractinian Corals
Source: PLoS One. 2016 Jan 11;11(1):e0147066. doi: 10.1371/journal.pone.0147066 (PMC4713449; doi:10.1371/journal.pone.0147066)
Supplement: S1 Fig — Microstructure of morphological “exceptions”: large and solitary zooxanthellate Cynarina lacrymalis (ZPAL H.25/43) (A) forms regular growth increments, whereas colonial and azooxanthellate Astroides calycularis (ZPAL H.25/58) (C) forms irregular bands. Exceptions from microstructural criterion: growth increments in skeleton of deep-water symbiotic coral Leptoseris fragilis (ZPAL H.25/48) (B) is not as regular as in many shallow-water zooxanthellate taxa, whereas in shallow-water (4–6m depth) asymbiotic Tubatraea tagusensis (ZPAL H.25/71) (D) banding pattern is clearly regular. Red arrows = regular bands, yellow arrows = irregular bands. SEM micrographs. (PDF) [file pone.0147066.s001.pdf]

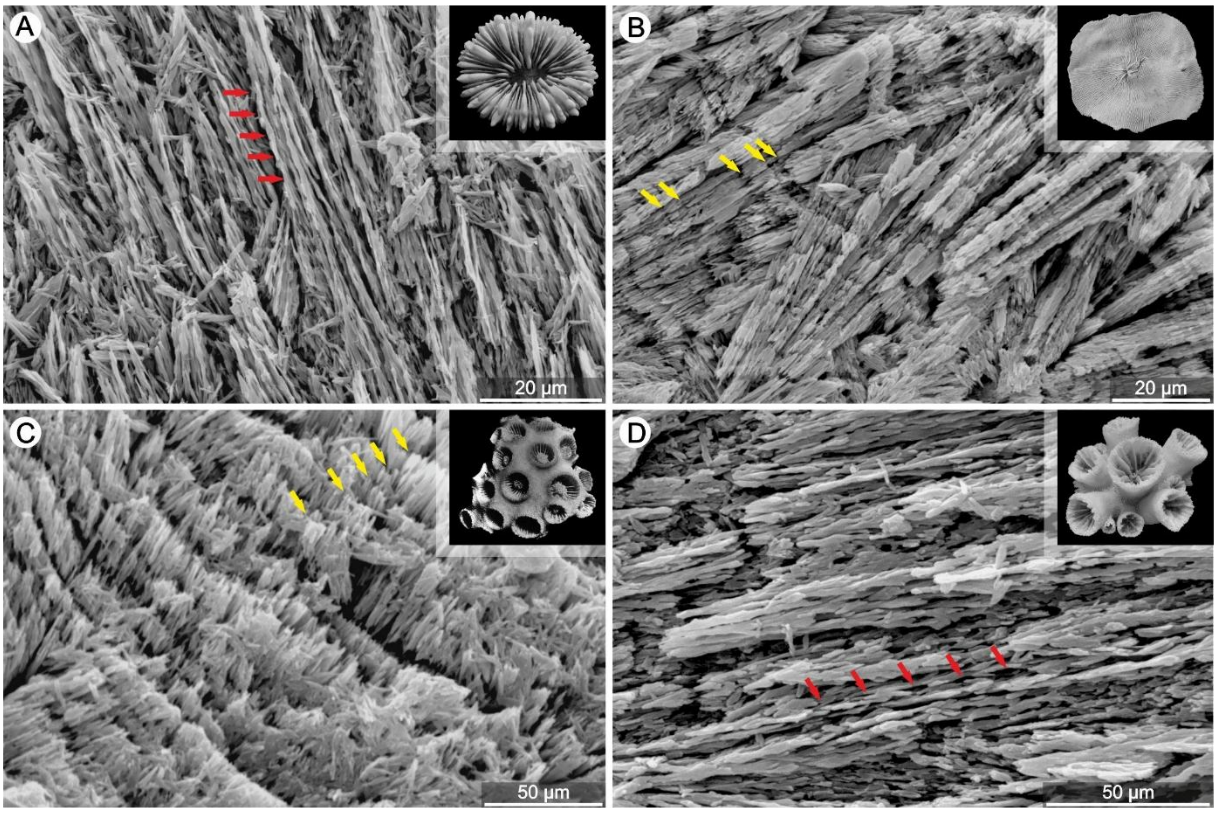

S1 Fig. Growth increments of TDs in some zooxanthellate (A,B) and azooxanthellate (C,D) corals whose morphological or microstructural features do not fit traditional criteria of symbiotic vs. non-symbiotic relationships. Microstructure of morphological "exceptions": large and solitary zooxanthellate *Cynarina lacrymalis* (ZPAL H.25/43) (**A**) forms regular growth increments, whereas colonial and azooxanthellate *Astroides calycularis* (ZPAL H.25/58) (**C**) forms irregular bands. Exceptions from microstructural criterion: growth increments in skeleton of deep-water symbiotic coral *Leptoseris fragilis* (ZPAL H.25/48) (**B**) is not as regular as in many shallow-water zooxanthellate taxa, whereas in shallow-water (4–6m depth) asymbiotic *Tubatraea tagusensis* (ZPAL H.25/71) (**D**) banding pattern is clearly regular. Red arrows = regular bands, yellow arrows = irregular bands. SEM micrographs.
