# Supplementary material for: Fine-Scale Skeletal Banding Can Distinguish Symbiotic from Asymbiotic Species among Modern and Fossil Scleractinian Corals
Source: PLoS One. 2016 Jan 11;11(1):e0147066. doi: 10.1371/journal.pone.0147066 (PMC4713449; doi:10.1371/journal.pone.0147066)
Supplement: S2 Fig — SEM photomicrographs of modern zooxanthellate species: (A) Madracis decactis (ZPAL H.25/52) and (B) Mussismilia hispida (ZPAL H.25/53) and azooxanthellate species: (C) Phyllangia americana (ZPAL H.25/69) and (D) Tubastraea tagusensis (ZPAL H.25/71). Regular growth increments are observed in zooxanthellate Mussismilia and azooxanthellate Tubastraea, and irregular growth increments in zooxanthellate Madracis and azooxanthellate Phyllangia. Skeletal characteristics of these corals, which differ from the typically pattern of banding (i.e. regular in zooxanthellate and irregular azooxanthellate corals), most likely reflects physiological peculiarities of Madracis (slow growth rate) and Tubastraea (relatively fast growth). Red arrows = regular bands, yellow arrows = irregular bands. (PDF) [file pone.0147066.s002.pdf]

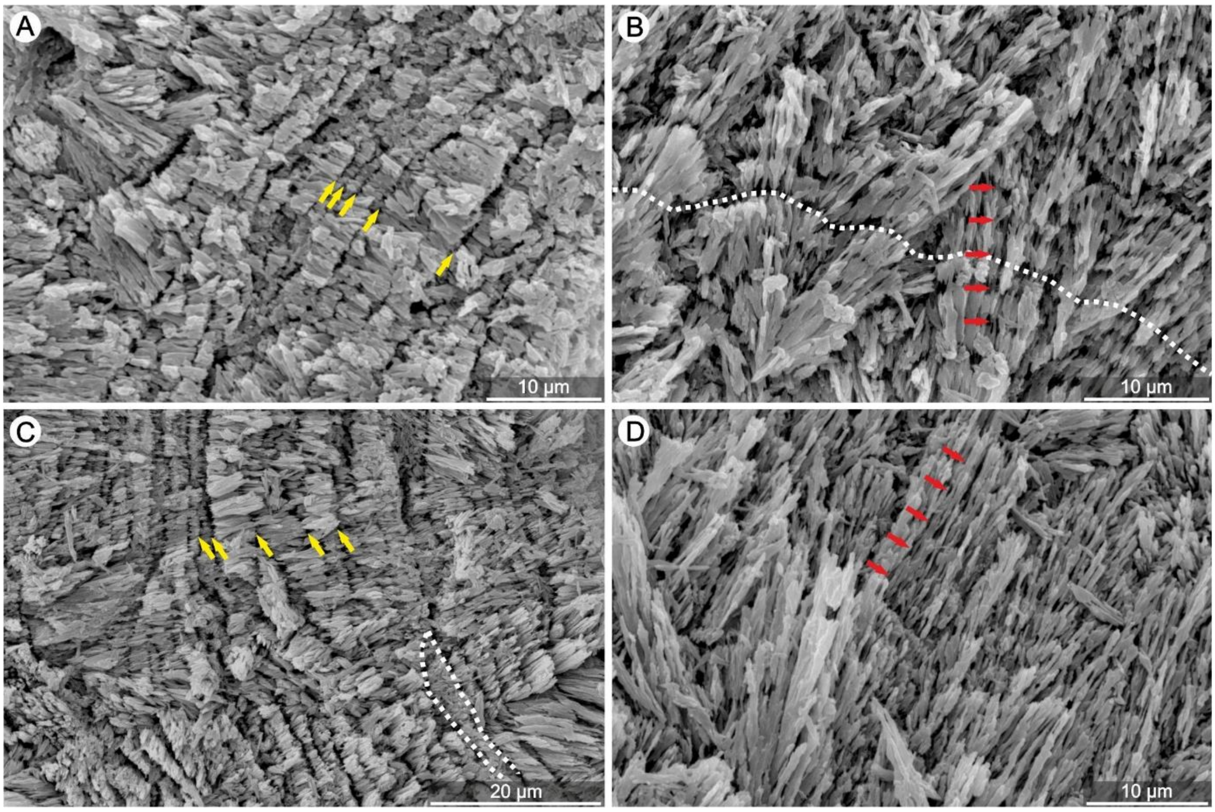

S2 Fig. Growth increments of TDs in zooxanthellate (A,B) and azooxanthellate (C,D) corals collected from the same site (Ilha dos Buzios, Brazil). SEM photomicrographs of modern zooxanthellate species: (**A**) *Madracis decactis* (ZPAL H.25/52) and (**B**) *Mussismilia hispida* (ZPAL H.25/53) and azooxanthellate species: (**C**) *Phyllangia americana* (ZPAL H.25/69) and (**D**) *Tubastraea tagusensis* (ZPAL H.25/71). Regular growth increments are observed in zooxanthellate *Mussismilia* and azooxanthellate *Tubastraea*, and irregular growth increments in zooxanthellate *Madracis* and azooxanthellate *Phyllangia*. Skeletal characteristics of these corals, which differ from the typically pattern of banding (i.e. regular in zooxanthellate and irregular azooxanthellate corals), most likely reflects physiological peculiarities of *Madracis* (slow growth rate) and *Tubastraea* (relatively fast growth). Red arrows = regular bands, yellow arrows = irregular bands.
