# Supplementary material for: Fine-Scale Skeletal Banding Can Distinguish Symbiotic from Asymbiotic Species among Modern and Fossil Scleractinian Corals
Source: PLoS One. 2016 Jan 11;11(1):e0147066. doi: 10.1371/journal.pone.0147066 (PMC4713449; doi:10.1371/journal.pone.0147066)
Supplement: S3 Fig — (A,A') Carbonic anhydrase. Expression of carbonic anhydrase is light-induced and its increased level during a day leads to increase of CO2 dehydration rate. High amount of bicarbonate ions delivered into calcification site together with increased activity of Ca2+-ATPase pump, results in enhanced skeleton growth. Conversely, low levels of this enzyme during the night restrict supply of HCO3-, that results in slower skeleton growth. (B,B’) Photosynthetic oxygen. During the day, oxygen released by photosymbionts is transported into calicoblasts where it enhances respiratory production of CO2 (later dehydrated by CA to bicarbonate ions) and ATP (used as calcium ion pump). High production of CO2 and ATP increases delivery of Ca2+ and HCO3- ions to calcification site and results in higher rate of calcification. At night, when photosynthetic O2 delivery is inhibited, respiratory production of CO2 and ATP is low. Consequently, this limits supply of Ca2+ and HCO3- ions and leads to decrease in growth rate. (C,C’) Photosynthetic hydrogen peroxide. H2O2 produced by zooxanthellae during photosynthesis cause lipid peroxidation of plasma membrane of calicoblasts thereby increasing leakage of Ca2+ ions into these cells. High concentration of calcium ions in calicoblasts results in their enhanced delivery into calcification site and higher growth rate. At night, when peroxide is not released, less Ca2+ ions is delivered to calicoblasts and calcification site, thus growth rate decrease. (D,D’) Metabolic phosphates. Phosphates produced in coral metabolism are regarded as inhibitors of skeletal growth. Their uptake by algae during photosynthesis cause that calcification process is not disturbed. When phosphates are not removed from the coral tissue during the night, they might reach calcification site and negatively affect deposition of carbonate. (E,E’) Hydroxyl ions. Photosynthesis-inducted secretion of hydroxyl ions in the coelenteron results in its alkalization and may facilitate [file pone.0147066.s003.pdf]

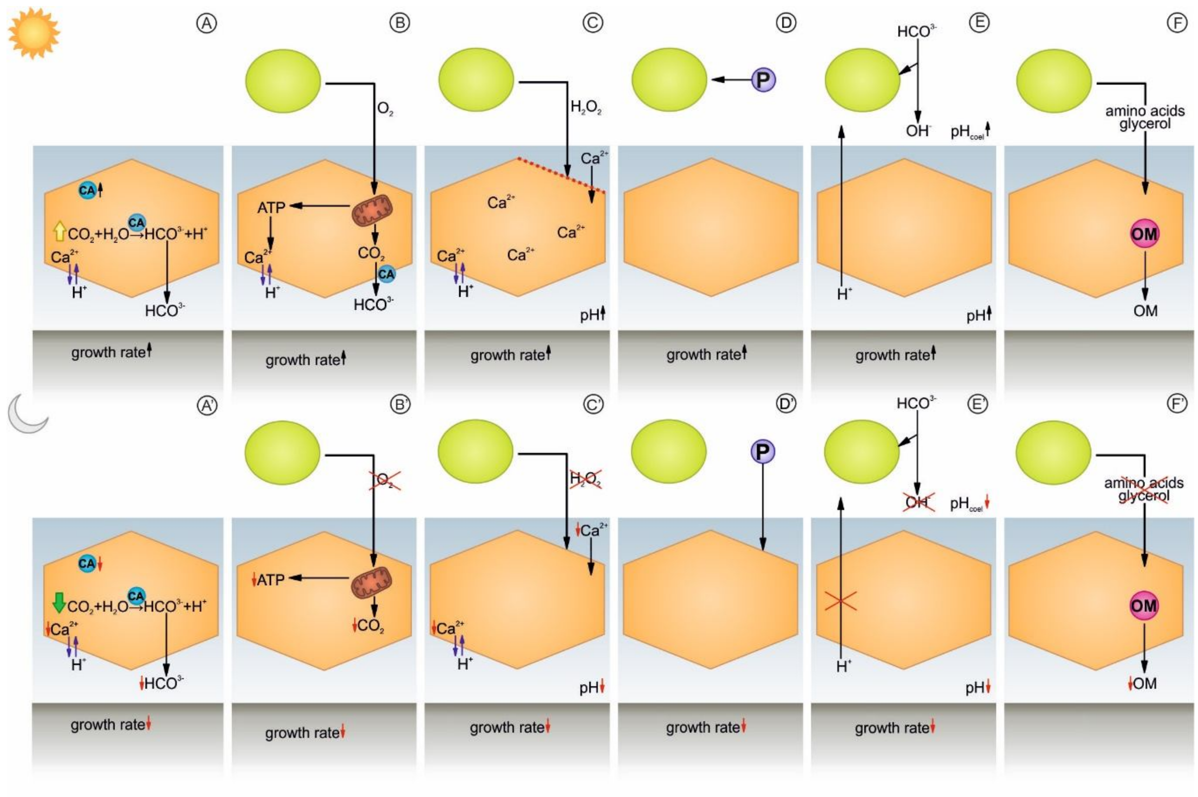

S3 Fig. Factors that potentially may induce day (A–F) – night (A'–F') differences in scleractinian coral biomineralization. (A,A') Carbonic anhydrase. Expression of carbonic anhydrase is light-induced and its increased level during a day leads to increase of CO_2 dehydration rate. High amount of bicarbonate ions delivered into calcification site together with increased activity of Ca^{2+}-ATPase pump, results in enhanced skeleton growth. Conversely, low levels of this enzyme during the night restrict supply of HCO_3^-, that results in slower skeleton growth. (B,B') Photosynthetic oxygen. During the day, oxygen released by photosymbionts is transported into calicoblasts where it enhances respiratory production of CO_2 (later dehydrated by CA to bicarbonate ions) and ATP (used as calcium ion pump). High production of CO_2 and ATP increases delivery of Ca^{2+} and HCO_3^- ions to calcification site and results in higher rate of calcification. At night, when photosynthetic O_2 delivery is inhibited, respiratory production of CO_2 and ATP is low. Consequently, this limits supply of Ca^{2+} and HCO_3^- ions and leads to decrease in growth rate. (C,C') Photosynthetic hydrogen peroxide. H_2O_2 produced by zooxanthellae during photosynthesis cause lipid peroxidation of plasma membrane of calicoblasts thereby increasing leakage of Ca^{2+} ions into these cells. High concentration of calcium ions in calicoblasts results in their enhanced delivery into calcification site and higher growth rate. At night, when peroxide is not released, less Ca^{2+} ions is delivered to calicoblasts and calcification site, thus growth rate decrease. (D,D') Metabolic phosphates. Phosphates produced in coral metabolism are regarded as inhibitors of skeletal growth. Their uptake by algae during photosynthesis cause that calcification process is not disturbed. When phosphates are not removed from the coral tissue during the night, they might reach calcification site and negatively affect deposition of carbonate. (E,E') Hydroxyl ions. Photosynthesis-inducted secretion of hydroxyl ions in the coelenteron results in its alkalization and may facilitate diffusion of protons from calcification site. Removal of H+ ions from calcification site may in turn lead to enhancement of skeletal growth. When photosynthesis is inhibited, low pH in the coelenteron inhibits protons diffusion resulting in drop of calcification rate. (F,F') Organic compounds. Zooxanthellae supply calicoblasts with organic matrix precursors (amino acids and glycerol), thereby increasing efficiency of skeleton formation during the day. During the night, when photosynthates delivery stops, secretion of OM and skeletal deposition decreases.
