# Supplementary material for: Fine-Scale Skeletal Banding Can Distinguish Symbiotic from Asymbiotic Species among Modern and Fossil Scleractinian Corals
Source: PLoS One. 2016 Jan 11;11(1):e0147066. doi: 10.1371/journal.pone.0147066 (PMC4713449; doi:10.1371/journal.pone.0147066)
Supplement: S1 Table — Inventory (1st column) and repository (2nd column) numbers, taxonomic attribution (3rd column) and available locality data (4, 5 columns including [year of collection] and {position on attached map}) of examined modern coral samples. The 6th column shows zooxanthellate (z) or azooxanthellate (az) status of the coral. Next columns include information about mean values (7th column), standard deviation (8th column) and coefficient of variation (9th column) of measured growth bands. (PDF) [file pone.0147066.s004.pdf]

S1 Table. Inventory (1st column) and repository (2nd column) numbers, taxonomic attribution (3rd column) and available locality data (4, 5 columns including [year of collection] and {position on attached map}) of examined modern coral samples. The 6th column shows zooxanthellate (z) or azooxanthellate (az) status of the coral. Next columns include information about mean values (7th column), standard deviation (8th column) and coefficient of variation (9th column) of measured growth bands.

1<br>Inventory number	2<br>Repository number ZPAL	3<br>Taxonomic attribution	4<br>Locality of the species treated in present study [collection year] {number on the map}	5<br>Depth range [m]	6<br>Symbiosis	7<br>Mean thickness of bands [µm]	8<br>Standard deviation	9<br>Coefficient of variation [%]
R-SCL046	H.25/42	*Acanthastrea echinata* (Dana, 1846) [Robusta: Lobophylliidae]	Pacific Ocean, Cook Islands (Mauke Island, Tukume) [1984] {1}	18-21	z	5	0.27	6
R-SCL085	H.25/43	*Cynarina lacrymalis* (Milne Edwards & Haime, 1849) [Robusta: Lobophylliidae]	North Pacific Ocean, Philippines, Sulu Archipelago, Tawitawi Islands, Sangasiapu Island [1908] {2}	16	z	3	0.32	9
R-SCL006	H.25/44	*Galaxea fascicularis* (Linnaeus, 1767) [Complexa: Oculiniidae]	North Pacific Ocean, Philippines, Southern Phil. Isl. [1887] {3}	few meters	z	3	0.31	12
R-SCL095A	H.25/45	*Goniastrea retiformis* (Lamarck, 1816) [Robusta: Merulinidae]	North Pacific Ocean, Mariana Islands, Saipan Island [1948] {4}	few meters	z	7	0.52	7
R-SCL095B	H.25/46	*Goniastrea retiformis* (Lamarck, 1816) [Robusta: Merulinidae]	North Pacific Ocean, Mariana Islands, Saipan Island [1948] {4}	few meters	z	3	0.30	9

R-SCL057	H.25/47	*Goniastrea stelligera* (Dana, 1846) [Robusta: Merulinidae]	Pacific Ocean, Great Barrier Reef, Lizard Island [1988] {5}	5-10	z	3	0.27	9
R-SCL456	H.25/48	*Leptoseris fragilis* Milne Edwards & Haime, 1849 [Complexa: Agariciidae]	Red Sea, off Eilat [1984]{6}	135	z	4	1.43	35
R-SCL007	H.25/49	*Lobactis scutaria* (Lamarck, 1801) [Robusta: Fungiidae]	Pacific Ocean, Great Barrier Reef, Lizard Island 14°68' S , 145°45' E [1980] {5}	< 5m	z	4	0.43	11
R-SCL049	H.25/50	*Lobophyllia hemprichii* (Ehrenberg, 1834) [Robusta: Lobophylliidae]	South Pacific Ocean, Papua New Guinea, Nagada Harbor [1992] {7}	few meters	z	4	0.46	13
R-SCL895	H.25/51	*Madracis decactis* (Lyman, 1859) [Complexa: Astrocoeniidae]	Atlantic Ocean, 23°47.437'S 45°08.653'W IIha dos Buzios, Brazil [2013] {8}	4-6	z	2	0.69	35
R-SCL090	H.25/52	*Merulina ampliata* (Ellis & Solander, 1786) [Robusta: Meruliniidae]	North Pacific Ocean, Micronesia, Caroline Islands, Pohnpei Islands, Ant Atoll, Nanpinapu [1984] {9}	few meters	z	5	0.75	15
R-SCL899	H.25/53	*Mussismilia hispida* (Verrill, 1901) [Robusta: Mussidae]	Atlantic Ocean, 23°47.437'S 45°08.653'W IIha dos Buzios, Brazil [2013] {8}	4-6	z	3	0.15	6

R-SCL486	H.25/16	*Pavona cactus* (Forskål, 1775) [Complexa: Agariciidae]	Indian Ocean, Yemen, Balhaf [2008]{10}	few meters	z	2	0.10	5
R-SCL1011	H.25/54	*Pocillopora damicornis* (Linnaeus, 1758) [Robusta: Pocilloporiidae]	Pacific Ocean, Hawaii, Kanehoe Bay, Coconut Island [2009]{11}	0.5-1	z	6	0.50	8
R-SCL480	H.25/55	*Porites porites* (Pallas, 1766) [Complexa: Poritidae]	Indian Ocean, off the coast of Kenya, near Watamu [1996]{12}	4	z	5	0.23	5
R-SCL053	H.25/56	*Symphyllia radians* Milne Edwards & Haime, 1849 [Robusta: Lobophylliidae]	Pacific Ocean, Caroline Islands, Pohnpei Islands, Pohnpei Island, Sokeh's Pass, Lagoon [1984]{9}	3.4	z	2	0.17	9
R-SCL032	H.25/57	*Symphyllia valenciennesii* Milne Edwards & Haime, 1849 [Robusta: Lobophylliidae]	North Pacific Ocean, Marshall Islands, Enewetak Atoll, Aniyaanii Island [1955] {13}	1-2	z	6	0.69	12
R-SCL419	H.25/58	*Astroides calycularis* (Pallas, 1766) [Complexa: Dendrophyllidae]	Mediterranean, Tunisia, off Tabarka [1969]{14}	14	az	3	1.36	39
R-SCL367	H.25/59	*Bathelia candida* Moseley, 1881 [Robusta: traditional Oculinidae]	Atlantic Ocean, Southern Argentina, San Jorge Gulf, 46°06.00'S 66°04.12'W [1961]{15}	103	az	8	4.23	55

R-SCL445/5	H.25/60	*Caryophyllia inornata* (Duncan, 1878) [Robusta: Caryophyllidae]	Mediterranean Sea, off Marseille, submarine cave [1960]{16}	15	az	3	2.99	88
R-SCL359	H.25/61	*Cyathelia axillaris* (Ellis & Solander, 1786) [Robusta: incertae sedis]	Red Sea/Gulf of Aden, 43°15.0'E 12°43.7'N [1987]{17}	228-235	az	5	2.94	58
R-SCL022A	H.25/62	*Desmophyllum dianthus* (Esper, 1794) [Robusta: Caryophyllidae]	Indian Ocean (NE St. Paul Island), MD50 cruise, Stat. 34/CP 152, 38°24.90'S, 77°25.10'E [1986]{18}	1050-1110	az	4	3.03	70
R-SCL022B	H.25/63	*Desmophyllum dianthus* (Esper, 1794) [Robusta: Caryophyllidae]	Indian Ocean (NE St. Paul Island), MD50 cruise, Stat. 34/CP 152, 38°24.90'S, 77°25.10'E [1986]{18}	1050-1110	az	1	0.59	60
R-SCL243	H.25/64	*Gardineria* sp. [Basalia: Gardineriidae]	Pacific Ocean (south of New Caledonia), 168°09.52'E/ 24°42.26'S [1986]{19}	230	az	2	0.90	42
R-SCL445/6	H.25/65	*Hoplangia durotrix* Gosse, 1860 [Robusta: traditional Caryophyllidae]	Mediterranean Sea, off Marseille, submarine cave [1960]{16}	15	az	2	0.91	43
R-SCL445/4	H.25/66	*Leptopsammia pruvoti* Lacaze-Duthiers, 1897 [Complexa: Dendrophylliidae]	Mediterranean Sea, off Marseille, submarine cave [1960]{16}	15	az	3	1.17	42

R-SCL082	H.25/67	*Lophelia pertusa* (Linnaeus, 1758) [Robusta: Caryophyllidae]	Atlantic Ocean, Blake Plateau, Off Jacksonville, Florida, 30°16.00'N 079°55.06'W [1965]{20}	494	az	1	0.70	56
R-SCL207	H.25/68	*Paracyathus pulchellus* (Philippi, 1842) [Robusta: traditional Caryophyllidae]	North Atlantic Ocean, Florida Keys, off Sand Key and Key West [1957]{21}	9-150	az	2	1.30	59
R-SCL905	H.25/69	*Phyllangia americana* Milne Edwards & Haime, 1849 [Robusta: traditional Caryophyllidae]	Atlantic Ocean, 23°47.437'S 45°08.653'W IIha dos Buzios, Brazil [2013]{8}	4-6	az	2	1.14	51
R-SCL029	H.25/70	*Stephanocyathus paliferus* Cairns, 1977 [Robusta: Caryophyllidae]	North Atlantic Ocean, off Venezuela, 9°45'N 59°47'W [1957] {22}	200-400	az	4	1.86	42
R-SCL906	H.25/71	*Tubastraea tagusensis* Wells, 1982 [Complexa: Dendrophyllidae]	Atlantic Ocean, 23°47.437'S 45°08.653'W IIha dos Buzios, Brazil [2013] {8}	4-6	az	3	0.18	6

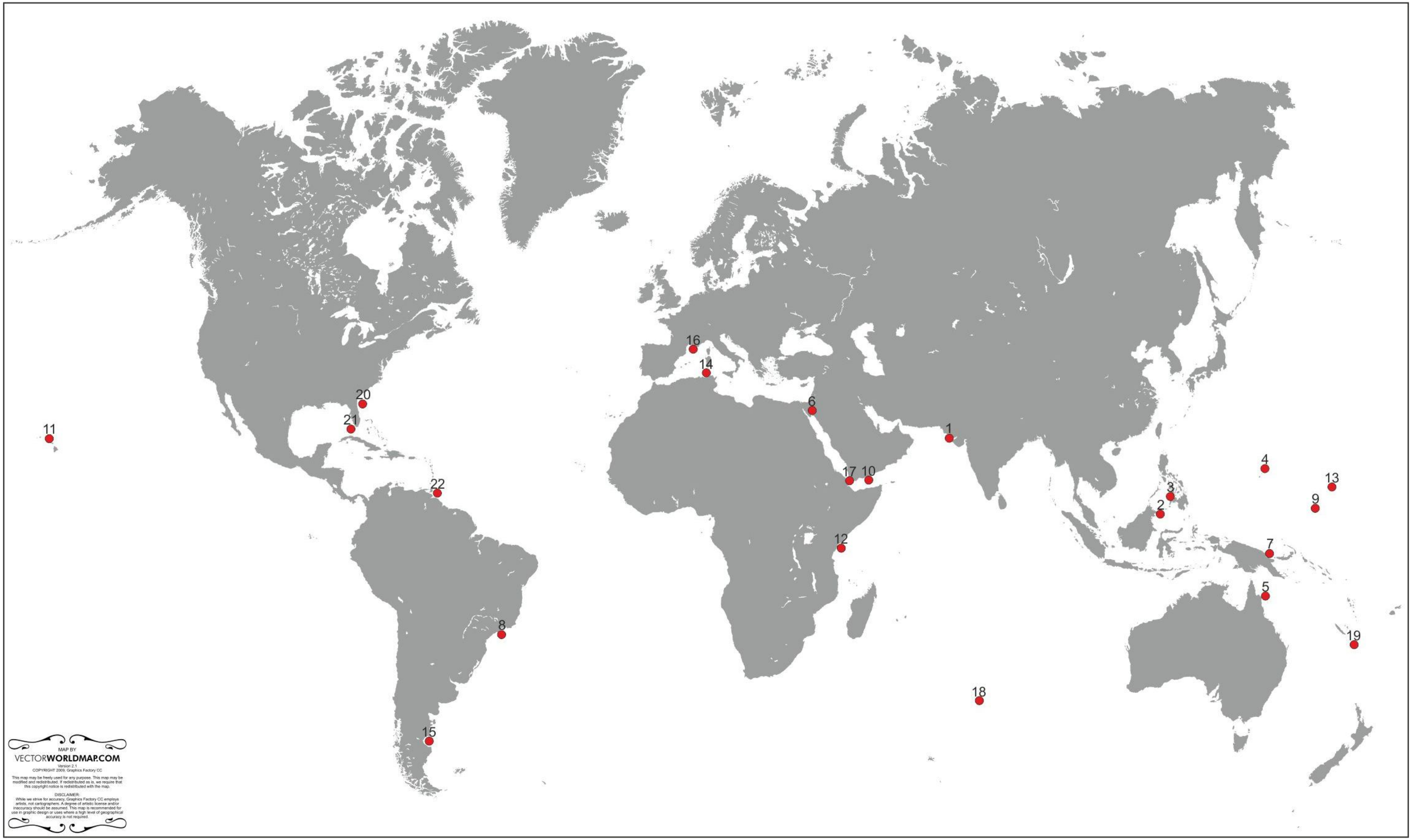
11
20
21
22
16
14
6
1
17
10
12
8
15
18
4
3
2
13
9
7
5
19
MAP BY
VECTORWORLDMAP.COM
Version 2.1
COPYRIGHT 2009, Graphics Factory CC
This map may be freely used for any purpose. This map may be modified and redistributed. If redistributed as is, we require that this copyright notice is redistributed with the map.
DISCLAIMER:
While we strive for accuracy, Graphics Factory CC employs artists, not cartographers. A degree of artistic license and/or inaccuracy should be assumed. This map is recommended for use in graphic design or uses where a high level of geographical accuracy is not required.
